# User Information Sharing and Hospital Website Privacy Policies

**DOI:** 10.1001/jamanetworkopen.2024.5861

**Published:** 2024-04-11

**Authors:** Matthew S. McCoy, Angela Wu, Sam Burdyl, Yungjee Kim, Noell Kristen Smith, Rachel Gonzales, Ari B. Friedman

**Affiliations:** 1 Department of Medical Ethics and Health Policy, University of Pennsylvania, Philadelphia; 2 Leonard Davis Institute of Health Economics, University of Pennsylvania, Philadelphia; 3 Carey Law School, University of Pennsylvania, Philadelphia; 4 Department of Emergency Medicine, University of Pennsylvania, Philadelphia

## Abstract

**Importance:**

Hospital websites frequently use tracking technologies that transfer user information to third parties. It is not known whether hospital websites include privacy policies that disclose relevant details regarding tracking.

**Objective:**

To determine whether hospital websites have accessible privacy policies and whether those policies contain key information related to third-party tracking.

**Design, Setting, and Participants:**

In this cross-sectional content analysis of website privacy policies of a nationally representative sample of nonfederal acute care hospitals, hospital websites were first measured to determine whether they included tracking technologies that transferred user information to third parties. Hospital website privacy policies were then identified using standardized searches. Policies were assessed for length and readability. Policy content was analyzed using a data abstraction form. Tracking measurement and privacy policy retrieval and analysis took place from November 2023 to January 2024. The prevalence of privacy policy characteristics was analyzed using standard descriptive statistics.

**Main Outcomes and Measures:**

The primary study outcome was the availability of a website privacy policy. Secondary outcomes were the length and readability of privacy policies and the inclusion of privacy policy content addressing user information collected by the website, potential uses of user information, third-party recipients of user information, and user rights regarding tracking and information collection.

**Results:**

Of 100 hospital websites, 96 (96.0%; 95% CI, 90.1%-98.9%) transferred user information to third parties. Privacy policies were found on 71 websites (71.0%; 95% CI, 61.6%-79.4%). Policies were a mean length of 2527 words (95% CI, 2058-2997 words) and were written at a mean grade level of 13.7 (95% CI, 13.4-14.1). Among 71 privacy policies, 69 (97.2%; 95% CI, 91.4%-99.5%) addressed types of user information automatically collected by the website, 70 (98.6%; 95% CI, 93.8%-99.9%) addressed how collected information would be used, 66 (93.0%; 95% CI, 85.3%-97.5%) addressed categories of third-party recipients of user information, and 40 (56.3%; 95% CI, 44.5%-67.7%) named specific third-party companies or services receiving user information.

**Conclusions and Relevance:**

In this cross-sectional study of hospital website privacy policies, a substantial number of hospital websites did not present users with adequate information about the privacy implications of website use, either because they lacked a privacy policy or had a privacy policy that contained limited content about third-party recipients of user information.

## Key Points

**Question:**

Do hospital websites include privacy policies that accurately disclose their use of third-party tracking technologies?

**Findings:**

In this cross-sectional analysis of a nationally representative sample of 100 nonfederal acute care hospitals, 96.0% of hospital websites transmitted user information to third parties, whereas 71.0% of websites included a publicly accessible privacy policy. Of 71 privacy policies, 40 (56.3%) disclosed specific third-party companies receiving user information.

**Meaning:**

These findings suggest that hospitals may not be presenting patients and other website users with adequate information about the privacy implications of website use.

## Teaser

This cross-sectional study examines whether hospital websites have accessible privacy policies and whether those policies contain key information related to third-party tracking.

## Introduction

Hospital websites are an essential resource for patients seeking health information and services. With a few clicks, a visitor to a hospital website can find a physician, schedule an appointment, view test results, or access reliable medical information. Yet along with these benefits come privacy risks for patients. In 2021, Mass General Brigham and the Dana Farber Cancer Institute reached an $18 million settlement with a class of plaintiffs who alleged that the hospital systems had used third-party tracking technologies on their public websites without seeking sufficient consent from users.^1^ Although the settlement was noteworthy, subsequent research has shown that hospital websites’ use of tracking technologies is commonplace.^2,3,4^

Privacy policies are often time-consuming to read and difficult to understand and, thus, provide an imperfect solution for protecting the privacy of hospital website users.^5,6,7^ Nonetheless, they serve important functions in the context of hospitals’ use of tracking technologies. Because hospitals risk regulatory scrutiny or civil lawsuits if they fail to adhere to the terms of their privacy policies, privacy policies can provide a mechanism for holding hospitals accountable for commitments to protect user privacy. Privacy policies also allow researchers, journalists, and consumer advocates to identify any discrepancies between disclosed and actual privacy practices. Finally, although most hospital website users may not read the privacy policy, the availability of a privacy policy respects individuals’ autonomy by giving them the ability to make better-informed decisions about whether and in what ways they choose to use a site.

Despite their importance, little is known about the availability or content of hospital website privacy policies. Although researchers have examined hospital websites, prior studies^8,9,10,11,12^ have focused on the content, accessibility, and usability of websites rather than their privacy policies. Conversely, there have been multiple studies^13,14,15^ of the privacy policies of health-related websites and applications, but these studies have not examined privacy policies of hospital websites despite the fact that these websites serve as an essential point of contact with the health care system. Building on prior work^2^ examining the prevalence of third-party tracking on hospital websites, the aims of this study were to determine, first, whether hospital websites have available privacy policies and, second, whether those policies contain information and are written in a way that would allow users to understand the types of personal information that the website may collect, potential third-party recipients of that information, and user rights with respect to tracking and data collection.

## Methods

### Study Population

This cross-sectional study did not include human participants and was, therefore, exempt from institutional review board review and the need for informed consent, in accordance with 45 CFR §46. We followed the Strengthening the Reporting of Observational Studies in Epidemiology (STROBE) reporting guidelines for cross-sectional studies.^16^

To construct a nationally representative sample of US hospitals, we identified all nonfederal acute care hospitals in the American Hospital Association database and their primary websites using an approach described in prior work.^2^ Consistent with prior methods, we excluded 47 hospitals for which a website could not be accessed.^2^ We then selected hospitals for privacy policy analysis via simple random sampling.

### Tracking Measurement

To determine the prevalence and characteristics of tracking across hospital websites, we visited the homepage of each hospital website using webXray,^17^ an open source, automated tool that detects third-party tracking code on webpages and that has previously been used in academic studies.^18,19,20,21^ We recorded the number of third-party data requests per page. These requests initiate data transfers, which typically include, at a minimum, a user’s internet protocol (IP) address and the URL (uniform resource locator) of the page being visited, to third-party domains—that is, domains other than that of the website the user is visiting. We also recorded the number of third-party cookies per page. Cookies are small pieces of code stored on a user’s browser that can serve as persistent identifiers, enabling third parties to track users across multiple sites. Tracking measurement and privacy policy retrieval and analysis took place from November 2023 to January 2024. As a robustness check, we compared webXray results for a random subsample of 30 study websites to the results browser-based tools Ghostery and Privacy Badger, which identify and block transfers to third-party domains.

### Privacy Polices

Privacy policies were independently obtained and analyzed by 2 reviewers (S.B. and Y.K.). Disagreements were resolved in weekly consensus meetings with the lead and senior author.

To obtain website privacy policies, we visually inspected the homepage of each website for links to a privacy policy, privacy statement, cookie statement, or other documents that might plausibly contain information related to user privacy. If we were unable to locate a privacy policy, we used the browser’s Find in Page functionality to perform a search for the word *policy* on the homepage. If we could not locate a privacy policy using these methods, we performed a Google search using the terms ([hospital name] AND *privacy policy*). Links to relevant documents were compiled for review.

We distinguished between website privacy policies and notice of privacy practice (NPP) documents according to their content, regardless of how they were labeled. A website privacy policy is a statement that describes how a website will collect, use, share, or sell data collected from users of the site, whereas an NPP describes how the institution will handle protected health information collected during clinical encounters and billing.

### Data Collection and Analysis

We collected data from privacy policies using a standardized data abstraction form. Drawing on prior studies of website privacy policies,^22,23,24,25^ we collected data in the following areas: information collected from website users (including both automatically collected and voluntarily provided information), uses of information collected from website users, third-party recipients of user information, user rights (such as a right to opt out of data collection), and any privacy protections for special populations.

In cases where a website had multiple relevant policy documents, documents were combined and treated as single policy for content analysis. In cases where a website combined an NPP and a privacy policy in a single document, we treated the document as a privacy policy and analyzed its contents using our standard approach.

Because length and complexity of privacy policies can be a barrier to user comprehension,^13,26^ we assessed the word count and readability of privacy policies, using document statistics in Microsoft Word version 16.69.1. Readability was estimated using both the Flesch-Kincaid Grade Level, which indicates a reading level by school grade according to the number of syllables per word and the average number of words per sentence, and the Flesch Reading Ease formula. Both scales have been validated in health care settings,^27,28^ are among the most commonly used measures of readability in the health care literature,^29^ and have been used in prior studies of privacy policy readability.^26^ Microsoft Word’s embedded Flesch-Kincaid Grade Level tool has been found to be more reliable than other automated readability tools that use the Flesch-Kincaid Grade Level.^30^ For websites that contained more than one document related to website privacy practices, we analyzed the reading level and word count of the document labeled *privacy policy*. For websites that combined an NPP and a privacy policy in a single document, we calculated word count and readability over the entire document.

### Statistical Analysis

We calculated descriptive statistics using Stata SE statistical software version 17.0 (StataCorp) and R statistical software version 4.2.3 (R Project for Statistical Computing) using 2-tailed 95% CIs and hypothesis tests. Statistical significance was set at *P* < .05. For comparison of the sample to the sampling frame of all nonfederal acute care hospitals, χ^2^ tests were used. For comparisons within the sample, survey statistics were used (R survey package version 4.2) to allow for finite population correction.^31^ For binary variables, the survey-weighted Rao-Scott scaled χ^2^ distribution for the loglikelihood from a binomial distribution was used. Where either exactly 0% or 100% of sampled privacy policies contained an element, the Clopper-Pearson exact 95% CI was used.

## Results

### Sample Characteristics

Table 1 compares the characteristics of the 100 hospitals included in the study sample with the characteristics of all nonfederal acute care hospitals included in the American Hospital Association database. The 100 hospitals included in the sample had 90 distinct websites. There were fewer websites than hospitals because some hospitals belonged to the same health system and shared a common website.

**Table 1.** Sample Characteristics Compared With All Nonfederal Acute Care US Hospitals^a^

Characteristic	Hospitals, No. (%)		*P* value^b^
	Study sample (n = 100)	All nonfederal acute care US hospitals (n = 3747)	
Region			
Northeast	15 (15.0)	452 (12.1)	.29
Midwest	19 (19.0)	816 (21.8)	
South	39 (39.0)	1657 (44.2)	
West	27 (27.0)	774 (20.7)	
Puerto Rico	0	48 (1.3)	
Profit status			
For profit	18 (18.0)	754 (20.1)	.62
Nonprofit	58 (58.0)	2275 (60.7)	
Public	24 (24.0)	714 (19.1)	
Unknown	0	4 (0.1)	
Part of a hospital system			
Yes	71 (71.0)	2434 (65.0)	.20
No	29 (29.0)	1313 (35.0)	
Medical school affiliation			
Yes	36 (36.0)	1199 (32.0)	.39
No	64 (64.0)	2548 (68.0)	
Size			
Small (<100 beds)	55 (55.0)	1814 (48.4)	.34
Medium (100-499 beds)	14 (14.0)	694 (18.5)	
Large (≥500 beds)	31 (31.0)	1239 (33.1)	

^a^ Excludes 47 hospitals for which a website could not be identified.

^b^ *P* values were calculated from the χ^2^ goodness-of-fit test.

### Tracking

We found that 96.0% (95% CI, 90.1%-98.9%) of hospital websites had at least 1 third-party data request and 86.0% (95% CI, 77.6%-92.1%) had at least 1 third-party cookie (eTable 1 in Supplement 1). Websites transferred user information to a median (IQR) of 9 (6-14) third-party domains and had a median (IQR) of 9 (3-16) third-party cookies (eTable 1 in Supplement 1).

We validated webXray output against 2 nonautomated, commercially available tools for a random subset of 30 hospital websites. For these websites, webXray recorded a median of 7 data transfers to third-party domains per website, Privacy Badger recorded a median of 7 with a correlation of 0.91 to webXray, and Ghostery recorded a median of 6, with a correlation of 0.84 to webXray.

### Policy Availability and Readability

Overall, 71 websites (71%; 95% CI, 61.6%-79.4%) had an accessible website privacy policy, of which 67 (67.0%; 95% CI, 57.3%-75.8%) were found via visual inspection and 4 (4.0%; 95% CI, 1.2%-9.1%) were found via Google search (Table 2). In addition, 69 websites (69.0%; 95% CI, 59.4%-77.6%) had a single privacy policy document, whereas 2 (2.0%; 95% CI, 0.3%-6.1%) divided information related to website privacy practices into 2 or more documents. In addition, 1 website (1.0%; 95% CI, 0.1%-4.4%) included only a document that was labeled as a privacy policy but actually was an NPP that contained no information regarding website privacy practices. Privacy policies were a mean length of 2527 words (95% CI, 2058-2997 words) and were written at mean Flesch-Kincaid Grade Level of 13.7 (95% CI, 13.4-14.1) and a mean Flesch Reading Ease score of 35.6 (95% CI, 33.9-37.2), which is considered difficult (Table 3).^28^

**Table 2.** Availability of Privacy Policies for Hospital Websites

Variable	Websites, No. (%) [95% CI] (n = 100)
Websites with a website privacy policy	71 (71.0) [61.6-79.4]
Single document	69 (69.0) [59.4-77.6]
Multiple documents	2 (2.0) [0.3-6.1]
Found via visual inspection	67 (67.0) [57.3-75.8]
Found via Google search^a^	4 (4.0) [1.2-9.1]
Found via browser search^b^	0 (0.0) [0.0-3.6]
Websites without a website privacy policy	29 (29.0) [20.6-38.4]
Notice of privacy practice mislabeled as privacy policy	1 (1.0) [0.1-4.4]
No privacy policy located	26 (26.0) [18.0-35.2]
Policy link broken	2 (2.0) [0.3-6.1]

^a^ Privacy policy was located using a Google search for the hospital name and *privacy policy*.

^b^ Privacy policy was located by searching within the page using the web browser’s Find in Page functionality.

**Table 3.** Length and Readability of 71 Hospital Website Privacy Policies

Variable	Mean (95% CI)
Word count	2527 (2058-2997)
Flesch-Kincaid Grade Level	13.7 (13.4-14.1)
Flesch Reading Ease	35.6 (33.9-37.2)

### Policy Content

Of 71 privacy policies, 69 (97.2%; 95% CI, 91.4%-99.5%) addressed types of user information automatically collected by the website (Table 4). The most common information types were IP address (57 policies [80.3%]), web browser name and version (53 policies [74.6%]), and the pages visited within the site (52 policies [73.2%]). In addition, 68 policies (95.8%; 95% CI, 89.3%-99.0%) addressed the collection of information voluntarily provided by users, including contact information (67 policies [94.4%]), name (62 policies [87.3%]), and demographic information (43 policies [60.6%]).

**Table 4.** Prevalence of Hospital Website Privacy Policy Statements Addressing User Information Collection

Variable	Hospitals, No. (%) [95% CI] (n = 71)
Privacy policy addresses automatically collected information	69 (97.2) [91.4-99.5]
Internet protocol address	57 (80.3) [69.9-88.5]
Web browser name and version	53 (74.6) [63.6-83.9]
Pages visited within the site	52 (73.2) [62.1-82.7]
Operating system name and version	44 (62.0) [50.2-72.9]
User behavior on site	40 (56.3) [44.5-67.7]
Date and time of visit	38 (53.5) [41.8-65.0]
Location data	27 (38.0) [27.1-49.8]
Duration of activity	22 (31.0) [20.9-42.5]
Terms used in site search engine	12 (16.9) [9.3-26.9]
Passwords	11 (15.5) [8.3-25.2]
Volume of data storage and transfers	1 (1.4) [0.1-6.2]
Privacy policy addresses voluntarily provided information	68 (95.8) [89.3-99.0]
Contact information	67 (94.4) [87.3-98.3]
Name	62 (87.3) [78.1-93.8]
Demographic information	43 (60.6) [48.8-71.6]
Financial and/or legal information	27 (38.0) [27.1-49.8]
Interests	24 (33.8) [23.4-45.4]

Nearly all policies, 70 of 71 (98.6%; 95% CI, 93.8%-99.9%), addressed purposes for which user information is collected (Table 5). Nearly three-quarters of policies (52 policies; 73.2%; 95% CI, 62.1%-82.7%) indicated that user information would be used for marketing and advertising purposes. A majority of policies (66 policies; 93.0%; 95% CI, 85.3%-97.5%) addressed third-party data recipients (Table 5). The most common categories of disclosed third-party recipients were service providers (50 policies [70.4%]), marketers and advertisers (27 policies [38.0%]), and subsequent firm owners (27 policies [38.0%]). Specific third-party companies receiving user data were named in 40 policies (56.3%; 95% CI, 44.5%-67.7%), with Google (35 policies [49.3%]) being the most common.

**Table 5.** Prevalence of Hospital Website Privacy Policy Statements Addressing Uses and Third-Party Recipients of User Information

Variable	**Hospitals, No. (%) [95% CI] (n = 71)**
Privacy policy addresses uses of user information	70 (98.6) [93.8-99.9]
Contact user regarding programs or services	62 (87.3) [78.1-93.8]
Track and analyze site use	61 (85.9) [76.4-92.8]
Provide information that may be of interest	57 (80.3) [69.9-88.5]
Provide marketing and advertising communications	52 (73.2) [62.1-82.7]
Improve experience as a user of hospital programs and services	49 (69.0) [57.5-79.1]
Manage programs and services	48 (67.6) [56.0-77.9]
Maintain and gain access to specially personalized areas of the site	37 (52.1) [40.4-63.7]
Prevent, detect, and investigate misuses	30 (42.3) [31.0-54.1]
Administer surveys or contests	25 (35.2) [24.6-46.9]
Verify user identity	23 (32.4) [22.1-44.0]
Auditing and security	13 (18.3) [10.4-28.5]
Process and ship requested and purchase products	11 (15.5) [8.3-25.2]
Maintain philanthropic endeavors and programs	7 (9.9) [4.3-18.3]
Manage business relationships	4 (5.6) [1.7-12.7]
Privacy policy address third-party data recipients	66 (93.0) [85.3-97.5]
Service providers	50 (70.4) [59.0-80.3]
Specific third-party company^a^	40 (56.3) [44.5-67.7]
Google/Alphabet	35 (49.3) [37.7-61.0]
Facebook/Meta	20 (28.2) [18.5-39.5]
X/Twitter	10 (14.1) [7.2-23.6]
Other named company^b^	7 (9.9) [4.3-18.3]
Marketing and advertising companies	27 (38.0) [27.1-49.8]
Buyers or successors in the event of a merger	27 (38.0) [27.1-49.8]
Contractors	23 (32.4) [22.1-44.0]

^a^ Subcategories sum to more than the total number of websites naming a specific third-party company because some privacy policies named more than 1 specific third-party company.

^b^ The number of website privacy policies naming a specific company other than Google, Facebook, or X. There were 9 companies mentioned by these 7 policies.

We found that 57 policies (80.3%; 95% CI, 69.9%-88.5%) addressed user privacy rights, the most common of which was the ability to disable site cookies (47 policies [66.2%]) and the ability to change or delete information collected by the website (34 policies [47.9%]) (eTable 2 in Supplement 1). In addition, 51 privacy policies (71.8%; 95% CI, 60.5%-81.5%) addressed privacy protections for special populations. All 51 of these policies addressed protections for children, and 2 (2.8%) also addressed protections for website users with disability.

## Discussion

In this cross-sectional study of a nationally representative sample of 100 nonfederal acute care hospitals, we found that although 96.0% of hospital websites exposed users to third-party tracking, only 71.0% of websites had an available website privacy policy. Polices averaged more than 2500 words in length and were written at a college reading-level. Given estimates that more than one-half of adults in the US lack literacy proficiency and that the average patient in the US reads at a grade 8 level, the length and complexity of privacy policies likely pose substantial barriers to users’ ability to read and understand them.^27,32^

When available, privacy policies frequently detailed the types of user information collected by the website and how that information might be used, but they were less informative with respect to specific third-party recipients of user information. Only 56.3% of policies (and only 40 hospitals overall) identified specific third-party recipients. Named third-parties tended to be companies familiar to users, such as Google. This lack of detail regarding third-party data recipients may lead users to assume that they are being tracked only by a small number of companies that they know well, when, in fact, hospital websites included in this study transferred user data to a median of 9 domains. Prior research^2^ has also shown that a wide range of companies commonly operate trackers on hospital websites, including data brokers and advertising companies with little or no consumer-facing presences.

In addition to presenting risks for users, inadequate privacy policies may pose risks for hospitals. Although hospitals are generally not required under federal law to have a website privacy policy that discloses their methods of collecting and transferring data from website visitors, hospitals that do publish website privacy policies may be subject to enforcement by regulatory authorities like the Federal Trade Commission (FTC).^33^ The FTC has taken the position that entities that publish privacy policies must ensure that these policies reflect their actual practices.^34^ For example, entities that promise they will delete personal information upon request but fail to do so in practice may be in violation of the FTC Act.^34^ In addition, as a contractual matter, website privacy policies can become legally binding documents, and breaches of such policies can elicit breach of contract claims under state law.^35^ Websites that collect specific categories of information from certain users may also be subject to other federal and state-specific requirements in terms of data collection and notice.^36^ Although the lawsuit against Mass General Brigham and the Dana Farber Cancer Institute was brought under Massachusetts law, plaintiffs have brought similar class action lawsuits in multiple states.^1^

### Limitations and Strengths

This study is limited by the fact that our manual search strategies may have failed to identify some website privacy policies and, thus, undercounted the number of available policies. However, because we systematically searched for policies using multiple methods, it is unlikely that typical website users would be able to find policies not identified in this study. We assessed policy readability using the Flesch-Kincaid Grade Level formula and the Flesch Reading Ease formula. Other readability formulas may generate different scores, although their outputs are generally well correlated.^37^ In addition, we were unable to determine the extent to which hospitals abide by key provisions in their privacy policies. We were limited by resources to evaluating only 100 hospital websites. However, because we used the American Hospital Association database as a sampling frame, the results are nationally representative within their calculated 95% CIs. Despite these limitations, our findings make a substantial contribution to the growing literature on hospital and other health care institutions’ use of tracking technologies on their websites by showing that a substantial number of hospital websites do not present users with adequate information about the privacy implications of website use, either because they lack a privacy policy or have a privacy policy that contains incomplete information about third-party tracking.

## Conclusions

To effectively protect user privacy, hospitals should carefully weigh the costs and benefits of including third-party trackers on their websites and should eliminate unnecessary third-party tracking technologies. They should also ensure that they have accessible and comprehensive privacy policies, which allow others to hold the hospitals accountable for their privacy practices and give users the resources they need to make informed decisions about website use.

## Notes

Supplement 1. **eTable 1.** Third-Party Tracking on Hospital Website Homepages **eTable 2.** Prevalence of Hospital Website Privacy Policy Statements Addressing Special Populations and User Rights

Supplement 2.
Data Sharing Statement
