# Supplementary material for: User Information Sharing and Hospital Website Privacy Policies
Source: JAMA Netw Open. 2024 Apr 11;7(4):e245861. doi: 10.1001/jamanetworkopen.2024.5861 (PMC11009820; doi:10.1001/jamanetworkopen.2024.5861)
Supplement: Supplement 1. — eTable 1. Third-Party Tracking on Hospital Website Homepages eTable 2. Prevalence of Hospital Website Privacy Policy Statements Addressing Special Populations and User Rights [file jamanetwopen-e245861-s001.pdf]

Supplemental Online Content

McCoy MS, Wu A, Burdyl S, et al. User information sharing and hospital website privacy policies. *JAMA Netw Open*. 2024;7(4):e245861. doi:10.1001/jamanetworkopen.2024.5861

eTable 1. Third-Party Tracking on Hospital Website Homepages

eTable 2. Prevalence of Hospital Website Privacy Policy Statements Addressing Special Populations and user Rights

This supplemental material has been provided by the authors to give readers additional information about their work.

© 2024 McCoy MS et al. *JAMA Network Open.*

eTable 1: Third-Party Tracking on Hospital Website Homepages

	**(N = 100)**
Homepages with a third-party request, No. (%) [95% CI]	96 (96.0) [90.0 – 98.9]
Homepages with a third-party cookie, No. (%) [95% CI]	86 (86.0) [77.6 – 92.1]
Third-party requests per homepage (Median, IQR)	9 (6 - 14)
Third-party cookies per homepage (Median, IQR)	9 (3 - 16)

© 2024 McCoy MS et al. *JAMA Network Open.*

eTable 2: Prevalence of Hospital Website Privacy Policy Statements Addressing Special Populations and user Rights

	**N=71**
	**No. (%) [95% CI]**
**Policy addresses user rights**	57 (80.3) [69.9 - 88.5]
Disabling site cookies	47 (66.2) [54.6 - 76.6]
Ability to change/delete information	34 (47.9) [36.3 - 59.6]
Right to access information	20 (28.2) [18.5 - 39.5]
Opting out of analytics collection	28 (39.4) [28.4 - 51.2]
Do Not Track functionality	14 (19.7) [11.5 - 30.1]
Right to opt out of sale of information	7 (9.9) [4.3 - 18.3]
Opting in or out of sharing location data	7 (9.9) [4.3 - 18.3]
Ability to clear location data	2 (2.8) [0.5 - 8.6]
Ability to change or disable location sharing functionality	2 (2.8) [0.5 - 8.6]
**Policy addresses privacy for any special population**	51 (71.8) [60.5 - 81.5]
Children*	51 (71.8) [60.5 - 81.5]
Children under 13	41 (57.7) [45.9 - 69]
Children under 18	11 (15.5) [8.3 - 25.2]
Disabled	2 (2.8) [0.5 - 8.6]

*Subcategories sum to more than 51 because some privacy policies included multiple provisions for different child age groups.

© 2024 McCoy MS et al. *JAMA Network Open.*
