# Supplementary material for: User Information Sharing and Hospital Website Privacy Policies
Source: JAMA Netw Open. 2024 Apr 11;7(4):e245861. doi: 10.1001/jamanetworkopen.2024.5861 (PMC11009820; doi:10.1001/jamanetworkopen.2024.5861)
Supplement: Supplement 2. — Data Sharing Statement [file jamanetwopen-e245861-s002.pdf]

Data Sharing Statement

McCoy. User Information Sharing and Hospital Website Privacy Policies. *JAMA Netw Open*. Published April 11, 2024. doi:10.1001/jamanetworkopen.2024.5861

Data
Data available: Yes
Data types: Data (not involving human participants)
How to access data: Data will be posted to figshare within one month of publication
When available: With publication

Supporting Documents
Document types: Statistical/analytic code
How to access documents: Code will be posted to figshare
When available: With publication

Additional Information
Who can access the data: General public
Types of analyses: Data on hospital privacy policies with hospital names and URLs removed
Mechanisms of data availability: Data will be posted to figshare
